# SGIP1 in axons prevents internalization of desensitized CB1R and modifies its function

**DOI:** 10.3389/fnins.2023.1213094

**Published:** 2023-07-20

**Authors:** Oleh Durydivka, Ken Mackie, Jaroslav Blahos

**Affiliations:** ^1^Institute of Molecular Genetics of the Czech Academy of Sciences, Prague, Czechia; ^2^Department of Psychological and Brain Sciences, Gill Center for Biomolecular Science, Indiana University, Bloomington, IN, United States

**Keywords:** cannabinoid receptor 1, synaptic transmission, axon enrichment, clathrin-mediated endocytosis, internalization

## Abstract

In the central nervous system (CNS), cannabinoid receptor 1 (CB1R) is preferentially expressed in axons where it has a unique property, namely resistance to agonist-driven endocytosis. This review aims to summarize what we know about molecular mechanisms of CB1R cell surface stability in axonal compartments, how these impact CB1R signaling, and to consider their physiological consequences. This review then focuses on a potential candidate for maintaining axonal CB1R at the cell surface, Src homology 3-domain growth factor receptor-bound 2-like endophilin interacting protein 1 (SGIP1). SGIP1 may contribute to the polarized distribution of CB1R and modify its signaling in axons. In addition, deletion of SGIP1 results in discrete behavioral changes in modalities controlled by the endocannabinoid system *in vivo*. Several drugs acting directly via CB1R have important therapeutic potential, however their adverse effects limit their clinical use. Future studies might reveal chemical approaches to target the SGIP1-CB1R interaction, with the aim to exploit the endocannabinoid system pharmaceutically in a discrete way, with minimized undesired consequences.

## Introduction

The Endocannabinoid System (ECS) is comprised of cannabinoid receptors, their endogenous ligands, *the endocannabinoids*, and the enzymes that synthesize and degrade endocannabinoids. Cannabinoid receptor 1 (CB1R), a G-protein coupled receptor (GPCR), is a core molecule of the neuronal ECS. In the central nervous system (CNS), CB1R is principally located presynaptically. It is found at the highest density on many GABAergic terminals but is also found at lower levels on some glutamatergic, cholinergic, serotonergic and noradrenergic terminals (Kano et al., 2009). CB1R is a major modulator of synaptic transmission, typically released in an activity-dependent fashion. Signaling via CB1R often affects higher order behaviors, including roles in controlling mood, fear extinction, addiction, and adaptive handling of stressful situations (Mechoulam and Parker, 2013; Micale et al., 2013; Lutz et al., 2015; Hebert-Chatelain et al., 2016; Morena et al., 2016). The ECS is also a promising target for pain treatments (Finn et al., 2021). Only a few drugs (dronabinol, nabilone, and nabiximols) targeting the ECS are on the market. Their indications are available at www.drugbank.ca.

Cell surface CB1R displays properties common to most GPCRs, with an important exception concerning its endocytosis properties in axons. Many GPCRs, including CB1R, form heterodimers with other GPCRs, and these interactions often alter the signaling properties of CB1R. CB1R heteromerization or signaling crosstalk can attenuate G protein activation, as in the case of μ opioid receptor activation in SK-N-SH cells and rat striatal membranes (Rios et al., 2006) and GABA_B_ receptor activation in rat hippocampal membranes (Cinar et al., 2008), while coexpression of D2-dopamine receptor in HEK293 cells reversed the effect of CB1R on cAMP production, leading to the increase in its production (Kearn et al., 2005). CB1R was shown to form macromolecular complexes with ghrelin GHS-R1a receptor in HEK293T cells, which impaired CB1R G_i_-mediated signaling (Lillo et al., 2021).

In addition to the usual GPCR-interacting proteins, several proteins appear to more specifically interact with CB1R and thus, to directly alter its signaling roles, as reviewed in (Fletcher-Jones et al., 2020). For example, CB1R interacting protein (CRIP1a) modulates the signaling of CB1Rs and its endocytosis (Stauffer et al., 2011; Blume et al., 2015; Mascia et al., 2017), Adaptor protein 3 (AP-3) plays a role in the processing and signaling of intracellular CB1R (Rozenfeld and Devi, 2008), while G-protein-associated sorting protein 1 (GASP1) controls lysosomal trafficking of phosphorylated and internalized CB1R (Martini et al., 2007; Niehaus et al., 2007; Rozenfeld and Devi, 2008; Mascia et al., 2017). Finally, SGIP1 hinders CB1R endocytosis by an unknown mechanism and considerably augments its cell surface stability (Hajkova et al., 2016). This association extensively regulates CB1R endocytosis with functional consequences in transfected cells (Hajkova et al., 2016; Gazdarica et al., 2022) and *in vivo* (Dvorakova et al., 2021).

## Limited CB1R internalization in axons and presynaptic boutons

In cultured neurons, CB1R is preferentially trafficked to axonal plasma membranes, including presynaptic boutons, where it accumulates. The neuronal plasma membrane is highly compartmentalized, which allows the directionality of information flow and concentration gradients of proteins and other molecules. The axon initial segment restricts the lateral diffusion of proteins and lipids between the somatodendritic and the axonal membrane. Synaptic active zone proteins and postsynaptic density proteins serve as anchors for synaptic machinery and receptors (Leterrier, 2018; Guzikowski and Kavalali, 2021). These barriers maintain protein localization and regulate their trafficking. The surface stability of CB1R expressed on axons is substantially greater than the stability of CB1R trafficked to other neuronal compartments or those expressed in heterologous systems.

CB1R agonist-driven endocytosis in transfected non-neuronal cell systems is rapid, with an internalization rate constant equal to 0.28 min^−1^ for agonist WIN55,212–2, while the constitutive internalization rate constant is 0.0032 min^−1^ (Zhu et al., 2019). Most of surface CB1R endocytosed within 30 min to 1 h (Rinaldi-Carmona et al., 1998; Jin et al., 1999). This contrasts with CB1R internalization from axons of cultured hippocampal neurons, where CB1R exhibits low levels of endocytosis following agonist treatment, remaining on the surface for up to 3 h. In this system, the removal of majority of the surface CB1R from axons was achieved only upon prolonged agonist exposure for 5–16 h (Coutts et al., 2001). No detectable change in CB1R staining intensity on the axonal surface was observed upon inverse agonist treatment in this study. Persistence of activated CB1R on the surface of axons contrasts with high rates of its internalization in somatodendritic compartments of rat primary hippocampal neurons (Leterrier et al., 2006). Treatment with the cannabinoid receptor high efficacy full agonist WIN55, 212–2 (WIN) rapidly reduced somatodendritic CB1R from the cell surface, followed by an increase in levels of CB1R-positive endosomes. In contrast, this study found that in axons, CB1R surface labeling persisted following the agonist treatment for 3 h due to reduced endocytosis. Importantly, incubation with the antagonist/inverse agonist AM281 resulted in a compartment-specific redistribution of CB1R on the neuronal surface. Blocking CB1R activity increases surface CB1Rs in the soma and dendrites after 3 h of treatment, but does not increase surface CB1Rs in axons. This increase in surface somatodendritic CB1R results from decreased endocytosis rate of the surface receptor due to CB1R inhibition. Thus, in compartments that have low levels of SGIP1, surface expression depends more on the receptor activity, desensitization and endocytosis.

Involvement of other endocytosis mechanisms was suggested in a report that described a discrepancy between rates of CB1R agonist-driven desensitization (loss of receptor responsiveness to agonist) and constitutive endocytosis in HEK293 cells and rat primary cerebral cortex neurons. The authors noticed that removal of cell surface CB1R involves both clathrin-mediated endocytosis (CME) and caveolae/lipid-rafts-dependent endocytosis (Wu et al., 2008). Authors of another study involving HeLa and Neuro-2a cells reported clathrin-dependent constitutive internalization of CB1R that is independent of beta-arrestin2 recruitment to the receptor (Gyombolai et al., 2013). Yet another report explored CB1R endocytosis independent of active state in rat primary hippocampal neurons (McDonald et al., 2007). In agreement with a previous study (Coutts et al., 2001), the authors conclude that the CB1R is delivered to the cell-surface membrane in both the axonal and somatodendritic compartments but reasoned that CB1R is removed from the somatodendritic plasma membrane by constitutive endocytosis. Agonist-driven endocytosis regulating CB1R levels on presynaptic bouton surface has been shown to rely on the agonist properties. Stochastic Optical Reconstruction Microscopy (STORM) imaging of slices of mouse hippocampal GABAergic interneurons demonstrated that chronic treatment with the phytocannabinoid Δ9-tetrahydrocannabinol (THC), the cannabinoid receptor partial agonist, enhanced CB1R internalization (Dudok et al., 2015). Similar results were obtained when the endogenous levels of cannabinoid receptor full agonist 2-arachidonoylglycerol (2-AG) were elevated by genetic deletion of its degrading enzyme, monoacylglycerol-lipase (MGL), in mouse enteric neurons (Taschler et al., 2015). In contrast to these findings, pharmacological inhibition of MGL by JZL184 to acutely elevate 2-AG levels did not increase the rate of CB1R internalization in hippocampal neurons (Thibault et al., 2013; Dudok et al., 2015; Lee et al., 2015).

Endocytic mechanisms of CB1R have been studied using an F238L mutation, which is a substitution of phenylalanine 238 in the transmembrane helix 4 of CB1R by leucine. This mutation promotes changes in the transmembrane regions associated with the activation of CB1R (Schneider et al., 2015). The mutated receptor in CB1R F238L mutant displays increased association with lipid rafts and elevated constitutive endocytosis in rat primary hippocampal neurons. Compared to wildtype CB1R, this mutant exhibited enriched axonal surface prevalence, together with reduced staining on the somatodendritic compartment surface (Wickert et al., 2018). Neuronal plasma membrane, particularly of axons and dendrites, contains a high proportion of ceramides, which decrease membrane fluidity. Synaptic membranes are enriched in cholesterol and sphingolipids and display lipid raft properties (Calderon et al., 1995; Saeedimasine et al., 2019; Fitzner et al., 2020; Westra et al., 2021).

Using single-quantum dot microscopy, Mikasova et al. examined the properties of CB1R on the surface of mouse primary cortical neurons (Mikasova et al., 2008). They found an immobile fraction of CB1R located in the vicinity of synapses that remained on the plasma membrane following at least 30 min of agonist stimulation. Therefore, in presynaptic compartments, a portion of CB1R is resistant to agonist-induced internalization and has low surface mobility. Together with reduced internalization, trapping of desensitized receptors in synaptic terminals by restricted lateral diffusion may be a complementary mechanism for fast regulation of presynaptic CB1R availability.

As stated above, removal of CB1R from the surface of the somatodendritic compartment by CME is relatively rapid (30 min – 1 h) (Leterrier et al., 2006). This resembles the properties of CB1R expressed in heterologous systems that are correspondingly regulated by CME (Rinaldi-Carmona et al., 1998; Jin et al., 1999). However, in axons, there is a pool of CB1R that is resistant to this fast endocytosis following receptor activation and a pool of CB1R that exhibits restricted lateral diffusion (Mikasova et al., 2008). In axons, slow removal of CB1R from the surface is likely mediated by different endocytic mechanisms over a longer time scale (16–20 h).

## CB1R sorting in neurons

CB1R distribution in neurons has a specific pattern, the receptor is enriched in the axons compared to the cell body and dendrites, where only a small portion of CB1R expressed by the cell is detected. This compartment-specific distribution may hypothetically be a result of two non-mutually-exclusive mechanisms. One possible mechanism is an active process that sorts synthesized CB1R to the axon, while the second is a process that preferentially stabilizes CB1R on the surfaces of axons. These mechanisms may work together, where active transport of CB1R is synergistically complemented by a mechanism that stabilizes CB1R in axons.

As discussed above, several studies have found that CB1R in neurons is stabilized on the surface of axons but not on the surface of somatodendritic compartments. By this premise, CB1R axonal enrichment would be achieved passively. In addition, active transport of CB1R to axons has been characterized in a study using an elaborate system allowing synchronized release of newly synthesized CB1R from the endoplasmic reticulum (ER) of rat primary hippocampal neurons (Fletcher-Jones et al., 2019). Shortly after release from the secretory compartment (25 min), CB1R reaches the cell surface in axons in amounts exceeding expression levels on the surface of other neuronal compartments.

The tetrameric adaptor protein 2 (AP-2) complex has been shown to play a role in the sorting of axonal proteins presynaptically. Loss of AP-2 impairs the localization of axonal, but not dendritic proteins in *Caenorhabditis elegans (C. elegans)* (Li et al., 2016). In this context, the AP-3 complex subunit delta was also identified as a CB1R-associated protein that plays an important role in the receptor’s trafficking, thus controlling the fate of intracellular CB1R. By utilizing HEK293 and Neuro2A cells and rat primary hippocampal culture as model systems, the authors show that the AP-3 complex may regulate the sorting of CB1R from the ER to axons. Downregulation of AP-3 delta results in an increased level of CB1R on the plasma membrane, likely due to its lowered association with endosomes. The impaired vesicle trafficking to presynaptic compartments results in an accumulation of the receptor on the somatodendritic plasma membrane. This finding is supported by the fact that AP-3 also exhibits polar distribution in neurons with enrichment in the axonal compartment and is involved in the sorting of other synaptic proteins (Salazar et al., 2004; Rozenfeld and Devi, 2008; Howlett et al., 2010; Fletcher-Jones et al., 2020).

Therefore, polarized CB1R distribution in neurons is achieved, or at least maintained by differential properties of internalization from surface of somatodendritic versus axonal compartments. Active axonal transport and maintenance of the polarity by stabilizing CB1R in axons may act synergistically with differential internalization. Note that the differential properties of internalization from the surface of the somatodendritic versus axonal compartments involve CME, but this might not be the only mechanism maintaining specific distribution of CB1R in neurons, as other types of internalization conceivably participate.

## CB1R desensitization and endocytosis

GPCR signaling is regulated by desensitization, which involves phosphorylation of serine and threonine residues in the intracellular C-terminus of CB1R by G protein-coupled receptor kinases (GRKs). This phosphorylation elicits arrestin recruitment to the GPCR that orchestrates a cascade of events culminating in receptor endocytosis.

Stimulation of CB1R activates diverse signaling pathways, including GRK2/3 activation (Jin et al., 1999; Nogueras-Ortiz and Yudowski, 2016) that mediate phosphorylation of serine/threonine residues of the CB1R C-terminus, leading to β-arrestin recruitment (Garcia et al., 1998; Moore et al., 2007; Al-Zoubi et al., 2019). The dynamics of the association between the receptor and its interacting partners involved in desensitization is controlled by the phosphorylation of two clusters of serine/threonine residues within the CB1R C-terminal tail. One cluster is between residues 425 and 429, namely ^425^SMGDS^429^, and another is between residues 460 and 468, ^460^TMSVSTDTS^468^ (human CB1R numbering). Both clusters were recognized as playing a role in β-arrestin2 recruitment (Hsieh et al., 1999; Jin et al., 1999; Bakshi et al., 2007; Daigle et al., 2008; Singh et al., 2011; Straiker et al., 2012; Morgan et al., 2014; Blume et al., 2017). In a recent study, we proposed that GRK3-mediated phosphorylation of these two regions has an opposing effect on the dynamics of GRK3-CB1R association (Gazdarica et al., 2022). While phosphorylation of residues within the ^460^TMSVSTDTS^468^ region favors this association, phosphorylation of residues within ^425^SMGDS^429^ region disfavors it. The spatial hindrance imposed by GRK3-mediated phosphorylation of ^425^SMGDS^429^ region may be the factor responsible for the dissociation of GRK3 from CB1R and thus allow β-arrestin2 to associate with CB1R. The application of GRK2/3-specific inhibitor cmp101 abrogated β-arrestin2 association with CB1R, which suggests that this β-arrestin2 association is dependent predominantly on GRK2/3-mediated phosphorylation of the CB1R C-terminus. In addition, GRK3 was shown to be predominantly involved in the desensitization of CB1R in HEK293 cells (Delgado-Peraza et al., 2016).

The binding of β-arrestin to the phosphorylated C-terminal tail of CB1R leads to a generally recognized series of consequences on the receptor and its signaling. First, β-arrestin occludes the G protein-interacting surface of CB1R, thus decreasing G protein-related signaling. Second, β-arrestin, as a scaffold protein, brings in proximity signaling components such as ERK1/2 and JNK cascades to the receptor, thus facilitating their activation. Third, receptor binding by β-arrestin exposes its AP-2-binding motif, thus initiating internalization of the receptor (Gurevich and Gurevich, 2015; Delgado-Peraza et al., 2016; Nogueras-Ortiz and Yudowski, 2016). A preference towards the G protein-or β-arrestin-related signaling is referred to as a biased signaling response and is a characteristic of a particular ligand (Leo and Abood, 2021).

Rapid internalization is typically seen following CB1R stimulation in heterologous transfected cell lines (Rinaldi-Carmona et al., 1998; Hsieh et al., 1999; Jin et al., 1999; Leterrier et al., 2004; Muro et al., 2010). The receptor internalization is mediated by the arrestin interaction with the receptor and consequent recruitment of the AP-2 complex. AP-2 is a central component of CME that assembles the cargo destined for endocytosis with clathrin and mediates interactions with plasma membrane lipids. Other proteins can also associate with, and control endocytic machinery. Important initiators of CME are muniscins, discussed below.

## Muniscins

The muniscin family of proteins comprises ubiquitously expressed FCH/F-BAR domain only proteins 1 and 2 (FCHO1/2) and SGIP1. FCHO1/2 are ubiquitously expressed, while SGIP1 expression is restricted mainly to neuronal tissue (Uezu et al., 2007). Muniscins interact with other molecules involved in the initiation of CME: endophilin (Trevaskis et al., 2005), intersectin (Dergai et al., 2010), and Eps15 (Uezu et al., 2007).

Several studies have proposed that FCHO1/2 and AP-2 have a special function in the initiation of CME (Hollopeter et al., 2014; Umasankar et al., 2014). One study in *C. elegans* showed that FCHO1/2 cooperate with AP-2 and promote the complex to reach its active conformation upon interaction within their AP-2 activating (APA) domain (Hollopeter et al., 2014). Interestingly, the APA domain is also found within the SGIP1 sequence. AP-2 recruitment to sites of a clathrin-coated pit (CCP) formation is thus assisted by preformed FCHO-Eps15 complexes. Conformational changes in the AP-2 complex unmasks its binding site for membrane lipids, namely phosphatidylinositol 4,5-bisphosphate (PIP2). The formation of FCHO–Eps15–AP-2 nanoclusters and AP-2–PIP2 interactions increases AP-2 residence time on the plasma membrane. While FCHO1/2 and Eps15 facilitate efficient AP-2 recruitment to the membranes, CME initiation is mostly dependent on the AP-2–PIP2 and AP-2 interactions with the arrestin recruited to the phosphorylated receptors (Ma et al., 2016).

A recent study examined AP-2 activation by its interaction with the muniscins. Distinct from the straightforward activation caused by the isolated interaction between AP-2 and FCHO1/2, the muniscins cooperate with the membrane to drive AP-2 into a new conformation that likely precedes cargo engagement (Partlow et al., 2022). The interaction with FCHO1/2, which possesses the F-BAR domain that interacts with and bends the membrane, and the membrane interaction of AP-2, emerges as indispensable to trigger the initial invagination of nascent endocytic pit (Partlow et al., 2022). However, it is not known if this conformational switch imposed on AP-2 by association with FCHO1/2, a process called priming, is also achieved by its interaction with SGIP1 (see below). Despite its high sequence similarity and shared domains, there is a major difference between SGIP1 and FCHO1/2 in their N-terminal regions. The F-BAR domain is present in FCHO1/2 proteins and was shown to initiate plasma membrane curvature via interactions with membrane lipids during the initial phases of CME. An unrelated membrane phospholipid-binding (MP) domain is present within the extreme SGIP1 N-terminus. The SGIP1 MP domain also interacts with membranes, however no structural data are available about this interaction.

## SGIP1 expression

SGIP1 is highly conserved across species, is expressed at high levels in the CNS (Uezu et al., 2007), and is enriched in domains adjacent to presynaptic boutons, in which it constitutes over 0.4% of the protein content (Wilhelm et al., 2014). Known physiological role of SGIP1 is its role in the regulation of energy homeostasis. SGIP1 mRNA levels are increased in the hypothalamus of the Israeli sand rat (*Psammomys obesus*), where they correlate with obesity of captive animals (Trevaskis et al., 2005). Furthermore, genetic variations of the SGIP1 gene are associated with disturbed energy balance in humans (Cummings et al., 2012). Interestingly, there is a putative association of SGIP1 mutations with neurological disorders in humans (Chwedorowicz et al., 2016).

In mice, there is significant overlap in the expression of SGIP1 and CB1R in many brain regions, particularly those involved in, for example, mood control, regulation of energy balance, nociception, and addiction. At the anatomical level, significant overlap between CB1R and SGIP1 is seen in prefrontal cortex, striatum, hippocampus, hypothalamus and pain processing circuits (Lein et al., 2007).

## SGIP1 interferes with CB1R internalization

In our earlier work, we showed that the interaction between SGIP1 and CB1R is independent of the receptor’s activation state. The interacting region of CB1R was determined as its C-terminus following helix 8. Moreover, phosphorylation of intracellular C-terminal residues in the region ^460^TMSVSTDTS^468^ of CB1R does not regulate this association. Therefore, the region of CB1R that could be involved in the interaction with SGIP1 may lie outside the phosphorylation motifs of the CB1R C-terminus. One prominent CB1R mutant that lacks helix 9 in its C-terminus (CB1RΔH9) resembles wildtype CB1R expressed in heterologous systems that lack SGIP1. This mutant has decreased cell surface stability, particularly on the axonal membrane in primary rat hippocampal neurons (Fletcher-Jones et al., 2019). The role of CB1R helix 9 in the interaction with SGIP1 should be addressed in future experiments. Other regions of CB1R, such as the outer surface of intracellular loop 3, might as well be involved in this interaction. The interaction of CB1R with SGIP1 hinders agonist-stimulated CB1R internalization as well as the internalization of the receptor in the absence of the agonist in transfected HEK293 cells (Hajkova et al., 2016).

Cellular processes facilitated by CB1R C-tail phosphorylation that would typically result in CME, are profoundly affected by SGIP1. SGIP1 expression results in elevated CB1R association with GRK3, and this impact is the greatest in later phases of CB1R desensitization (Gazdarica et al., 2022). Arrestin interaction with CB1R is also elevated in presence of SGIP1 and persists longer than it does in absence of SGIP1 (Hajkova et al., 2016). SGIP1 presence reduces ERK1/2 signaling by CB1R (Hajkova et al., 2016), likely as the consequence of impaired activation of the arrestin-mediated ERK1/2 pathway as SGIP1 prevents CB1R internalization. The effect of SGIP1 on CB1R signaling in transfected HEK293 cells was probed using two agonists, 2-AG and WIN55,212–2 (WIN) (Hajkova et al., 2016). WIN-stimulated arrestin recruitment and activation of ERK1/2 signaling were greater than that stimulated by 2-AG. SGIP1 substantially suppressed ERK1/2 signaling by WIN-activated CB1R, compared to the situation when 2-AG was applied. This suggests that, if low levels of arrestin are recruited to the CB1R and during less efficacious signaling of ERK1/2, SGIP1-mediated effects are less profound. However, when arrestin is robustly recruited to the receptor, and ERK1/2 pathway activation is strong, the role of SGIP1 is superior.

**Figure 1:**
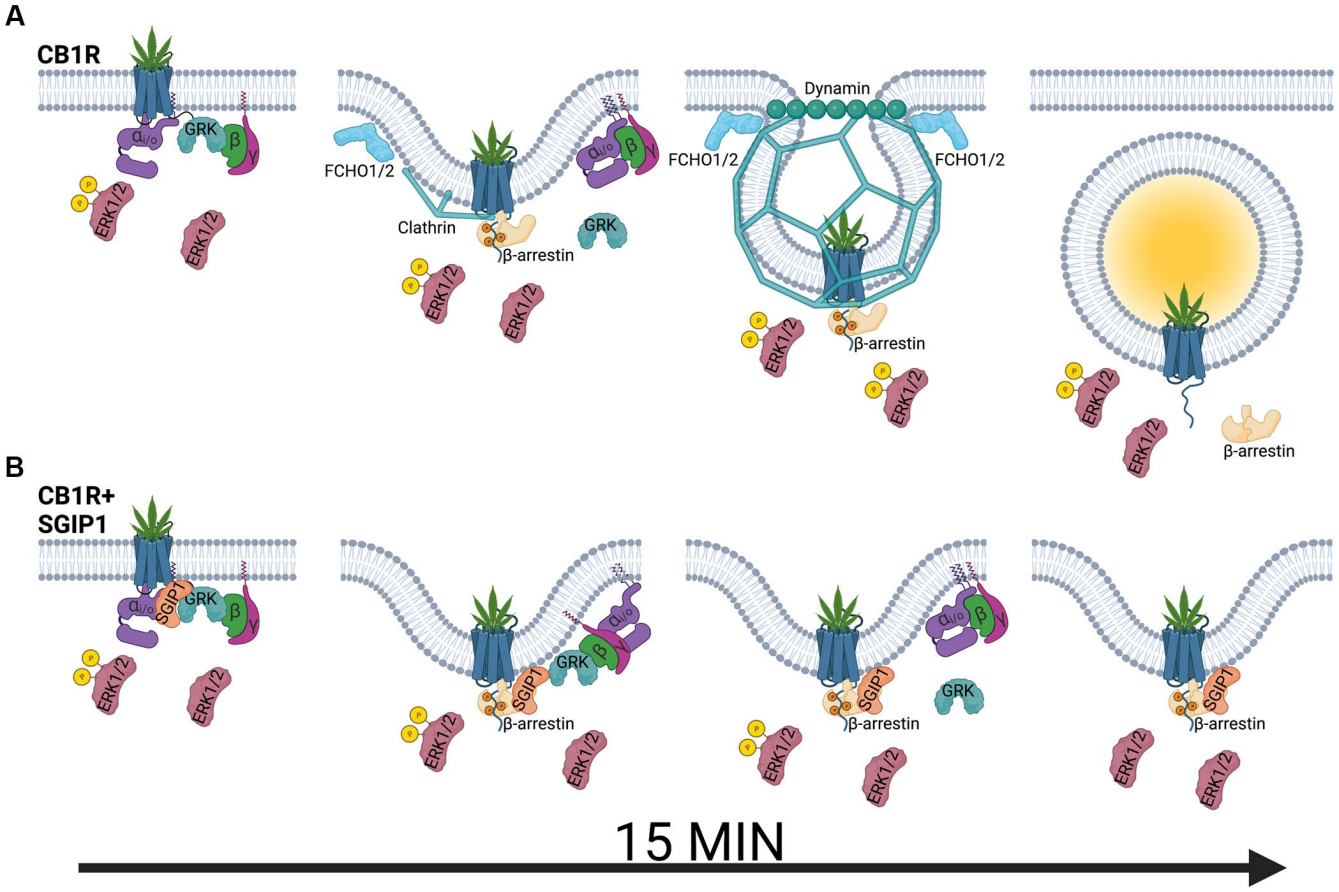
Modulation of cannabinoid receptor 1 signaling by SGIP1. **(A)** Upon agonist binding, cannabinoid receptor 1 (CB1R) activates heterotrimeric G_i/o_ proteins, composed of α_i/o_, β, and γ subunits, which activate signaling cascades such as mitogen-activated protein kinase ERK1/2 or modulate activity of ion channels. At the same time, G protein-coupled receptor kinases (GRK) phosphorylate the active receptor, increasing its affinity for β-arrestins. β-arrestins desensitize the receptor and promote its clathrin-mediated internalization. ERK1/2 phosphorylation is also mediated by β-arrestin-mediated pathways, including signaling from the endocytosed receptor pool. **(B)** ERK1/2 signaling is diminished in the presence of SGIP1. Therefore, SGIP1 association with CB1R decreases ERK1/2 phosphorylation, likely from the lack of signaling from the endocytosed pool of CB1R. The endocytic machinery includes FCH/F-BAR domain only protein 1 and 2 (FCHO1/2), which initiate membrane invaginations, clathrin and its adaptor protein 2 (AP-2, not shown), which stabilize the endocytic pits and link the receptor to the endocytic pits, and dynamin, which pinches off the invaginated pits. SGIP1 is an endocytic protein that interferes with CB1R endocytosis by an undescribed mechanism. As a result of inhibited CB1R endocytosis, the persistence of the desensitized receptor on the cell surface increases and prolongs GRK and β-arrestin association with the receptor.

GPCRs may elicit their signaling from different cell compartments in waves; this could further underline the role of CB1R-SGIP1 interactions in the dynamics of interaction within the signalosome as CB1R desensitize. The following recognized model suggests two distinct waves of GPCR signaling: one wave arises from the activated GPCRs on the cell surface, while the second wave arises from the internalized GPCRs as they pass through various intracellular compartments (Daaka et al., 1998). Agonist stimulation of CB1R on the plasma membrane results in the activation of G proteins, mainly G_i/o_, resulting in ERK1/2 activation, and phosphorylation of the receptor by GRK2/3, which is coupled with subsequent binding of β-arrestins to the receptor (Figure 1A). Pertussis toxin (PTX) blocks G_i/o_ signaling, therefore ERK1/2 activation by G_i/o_-mediated pathway is obliterated. However, as GRK is not activated, β-arrestin recruitment to the receptor does not occur. Both pathways leading to the phosphorylation of ERK1/2 are blocked by PTX. β-arrestins inhibit the receptor’s ability to activate G proteins and promote internalization of the receptor. During internalization, CB1R becomes uncoupled from G proteins because of incorporation into endosomes; however, it is still bound, albeit transiently, by β-arrestins. Apart from desensitization, β-arrestins can also promote activation of particular pathways, such as ERK1/2. Therefore, ERK1/2 activity integrates signaling inputs from CB1R both on the plasma membrane and in endosomes.

The following hypothesis that aims at understanding the relationship between SGIP1 and CB1R and how this relationship affects events following CB1R desensitization suggests: During CB1R desensitization, β-arrestins are recruited to and interact with the phosphorylated CB1R leading to its internalization. Internalization terminates the transient association between phosphorylated CB1R and β-arrestins. The presence of SGIP1 stalls CB1R internalization. Therefore, in the presence of SGIP1, GRK2/3 and β-arrestin interactions with the desensitized CB1R persist longer (Figure 1B). The persistent CB1R-β-arrestin interaction leads to alterations of CB1R signaling profile, such as an inhibition of ERK1/2 activity.

Although it remains to be fully elucidated how SGIP1 opposes CB1R internalization, two (so far hypothetic) mechanisms may be involved. FCHO1/2 from the muniscin family is known to initiate the invagination of the plasma membrane as CME is initiated, resulting in cargo internalization, while, in case of CB1R, SGIP1 opposes this process. The apparent difference between the organization of the N-terminal domains of SGIP1 and FCHO1/2 may explain their differing effects on CB1R. In FCHO1/2, the folding of the N-terminal portion forms F-BAR domains, which initiate bending of the membrane to form CME pits (Henne et al., 2007). In contrast, the N-terminus of SGIP1 contains the MP domain with no sequence similarity with the F-BAR domain (Uezu et al., 2007). Thus, based on this structural difference, the MP and F-BAR domains interact differently with the plasma membrane. SGIP1 may not be involved in initiation of nascent pit formation. Hypothetically, it may hinder this process and stall it, if it replaces FCHO1/2 proteins.

Another, not necessarily concurrent, hypothesis that may explain SGIP1’s hindrance of CB1R endocytosis reflects the dynamics of muniscin involvement in CME. FCHO1/2 proteins, following the early stage of endocytosis, are partitioned on the edge of the growing nascent endocytic pit and are excluded from mature vesicles (Henne et al., 2007; Ma et al., 2016; Sochacki et al., 2017). Due to persistent interactions between SGIP1 and CB1R, SGIP1 is present in the nascent pit during later stages of endocytic pit formation. As a result, CME ceases at the stage of intermolecular interactions involving lipids from the membrane, SGIP1, and the AP-2 complex. Clearly, more experimental data are needed to differentiate between these two hypotheses.

Given these open questions, we examined regulation of CB1R signaling in the presence of SGIP1 using transfected cells. These experiments revealed profound effects of SGIP1 on CB1R signaling in heterologous systems. Interestingly, specific dwell times of CB1R during CME for 2-AG and WIN have been shown to influence signaling properties in transfected HEK293 cells (Flores-Otero et al., 2014). However, SGIP1 imposes a more pronounced impact on CB1R signaling upon co-transfection with the receptor in this system.

**Figure 2:**
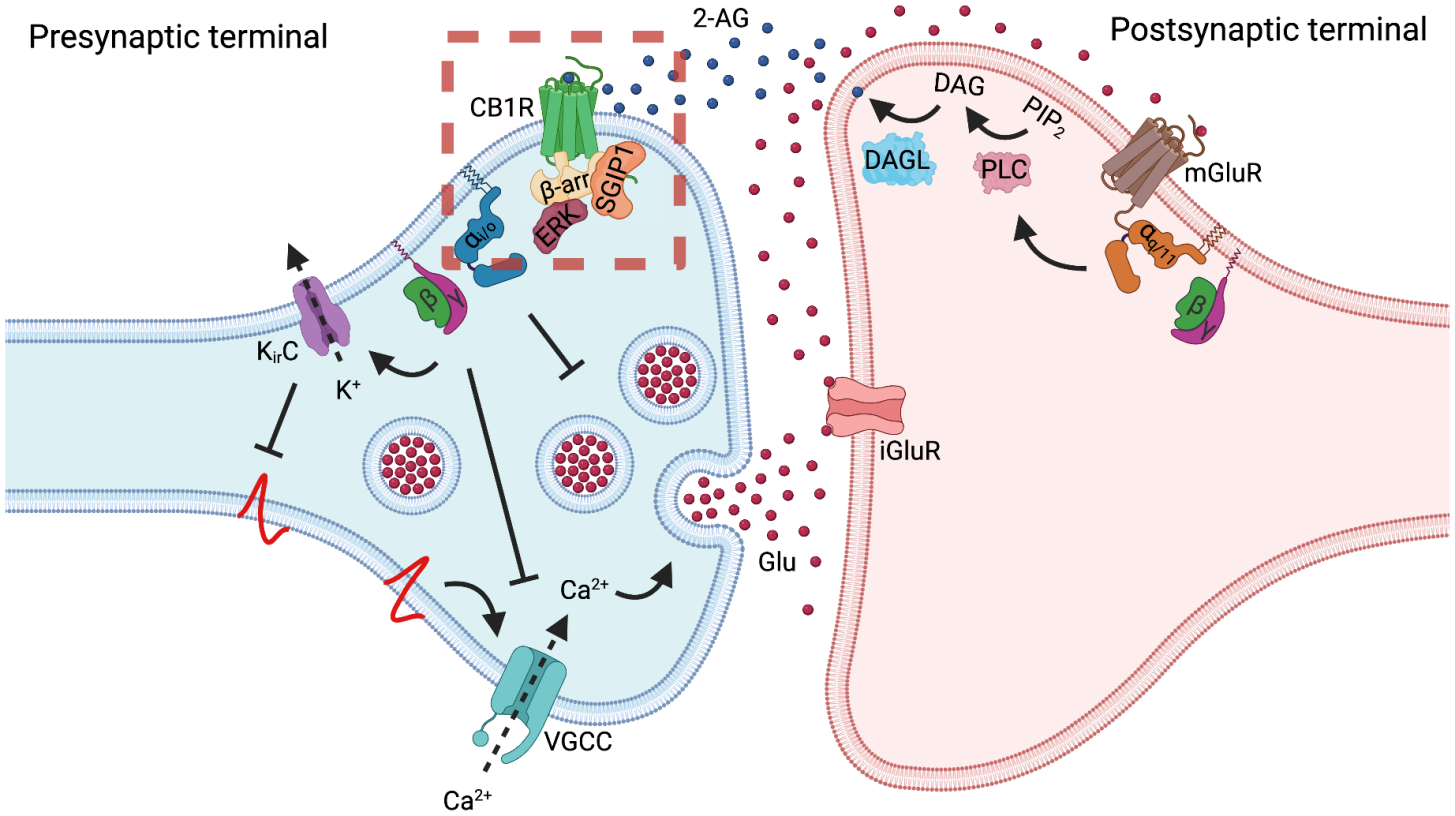
Cannabinoid receptor 1 regulates synaptic transmission. In the case of a glutamatergic synapse, depolarization of the presynaptic membrane results in the release of glutamate (Glu) that activates postsynaptic ionotropic glutamate receptors (iGluR) and, especially in situations of massive release, metabotropic glutamate receptors 1 and 5 (mGluR). G_q/11_ protein-mediated signaling cascade of mGluR activates phospholipase C (PLC), converting phosphatidylinositol 4,5-bisphosphate (PIP_2_) to diacylglycerol (DAG) and inositol 1,4,5-trisphosphate (IP_3_, not shown). Postsynaptically-located diacylglycerol lipase (DAGL) converts DAG into 2-arachidonoylglycerol (2-AG). 2-AG diffuses to the presynaptic membrane, where it activates cannabinoid receptor 1 (CB1R). Often, this regulation is referred to as the on-demand production of 2-AG. Another endocannabinoid, N-arachidonoylethanolamine (AEA), which is perhaps produced tonically, also activates CB1R (not shown). Activation of CB1R results in a decrease in synaptic vesicle exocytosis by several mechanisms. Inhibition of synaptic vesicle exocytosis can be mediated through G_i/o_ activity as well as through G_βγ_-mediated inhibition of voltage-gated calcium channels (VGCC) and activation of inwardly-rectifying potassium channels (K_ir_). Inhibition of VGCC decreases the levels of Ca^2+^ in the presynapse, thus inhibiting vesicle exocytosis. Activation of K_ir_ channels hyperpolarizes presynaptic membrane, thus preventing activation of VGCC. Increased release of glutamate elevates production of endocannabinoids, which in turn activate CB1R. Increased CB1R activity inhibits vesicle exocytosis and the glutamate release; thus, CB1R plays a role as a negative feedback loop regulator of synaptic transmission, contributing to synaptic plasticity. While agonist-activated CB1R is rapidly endocytosed from the dendrosomatic neuronal surface, presynaptically-located CB1R displays a high degree of membrane stability, both at a basal state and upon agonist activation. This stabilization may be the result of SGIP1 interaction with the receptor, which inhibits the receptor’s internalization or lateral mobility. The dashed red square indicates the signaling aspects of the CB1R-SGIP1 relationship during the initial phase of desensitization tested in heterologous systems and depicted in Figure 1.

CB1R, as a part of the ESC, is involved in tight retrograde regulation of synaptic transmission. One of the most important consequences of presynaptic CB1R activation in the CNS is the inhibition of neurotransmitter release into the synaptic cleft (Ohno-Shosaku and Kano, 2014). Glutamate release into the synaptic cleft depolarizes the postsynaptic membrane by opening ionotropic receptors as well as stimulates production and mobilization of endocannabinoids by activating metabotropic receptors (Figure 2). Activated CB1R decreases the probability of glutamate release by several mechanisms, such as inhibition of synaptic vesicle endocytosis, inhibition of voltage-gated calcium channels (VGCC), and activation of inwardly-rectifying potassium channels (K_ir_).

The data discussed above demonstrate that in heterologous systems, SGIP1 stabilizes the activated CB1R on the plasma membrane, which influences CB1R signaling cascades. By applying these consequences to a homogenous system such as a synapse, we may speculate that SGIP1 can stabilize the activated CB1R on the presynaptic membrane by obstructing CB1R removal from the membrane or by restricting the receptor’s lateral mobility in the membrane (Figure 2). The retention of CB1R on the presynaptic membrane would amplify the signaling of the receptor so that the receptor would be readily available after it was desensitized by β-arrestin binding. Therefore, SGIP1 might be the factor responsible for differential endocytosis of CB1R from axonal and somatodendritic membranes in primary neuronal cultures, discussed earlier. This assumption should be closely evaluated in assays employing primary neurons and by applying *in vivo* approaches. To better understand the role of the CB1R-SGIP1 relationship *in vivo*, genetically modified mice lacking SGIP1 were evaluated in several tasks in which the ECS modulates behaviors.

SGIP1 knockout (SGIP1−/−) mice were used to investigate the *in vivo* role of SGIP1. Sensorimotor processing, mobility and exploratory drive, and working memory were not altered in SGIP1−/− mice. However, SGIP1−/− mice were noted to have specific abnormalities in an interesting subset of behaviors, specifically in tests that examine domains of mood-related behaviors and nociception. Interestingly, SGIP1 deletion did not decrease body weight; thus, it appears that only upregulation of hypothalamic SGIP1 is associated with obesity (Trevaskis et al., 2005).

The ECS has a central role in mood, fear, and accommodation to stressful situations [reviewed in (Mechoulam and Parker, 2013; Micale et al., 2013; Lutz et al., 2015; Morena et al., 2016)]. SGIP1−/− mice have altered mood-related behaviors and emotionality. Behavioral tests that exploit the natural conflict between the drive to explore a novel environment and the tendency to avoid areas of high illumination exposed specific consequences of SGIP1 absence. SGIP1−/− males showed less thigmotaxis in the open field (OF) arena compared to WT males, while no differences were seen in female SGIP1−/− and WT mice. In the elevated plus maze (EPM) test assessing anxiety-like behaviors, both male and female SGIP1−/− mice had longer open arms times and traveled a greater distance in the EPM than did WT mice. As assessed in the tail suspension test (TST) SGIP1−/− mice showed greater resilience. These findings imply that deletion of SGIP1 causes an anxiolytic-like phenotype. The magnitude of these effects varied between sexes. The anxiolytic-like phenotype of SGIP1−/− and higher resistance to an unescapable situation complement pharmacological studies that found anxiolytic-like and antidepresive-like effects of enhanced endocannabinoid transmission by inhibition of endocannabinoid degradation (Bortolato et al., 2007; Danandeh et al., 2018). Extinction of fear memories is modulated by the ECS (Marsicano et al., 2002; Lutz et al., 2015). Aversive memories were extinguished similarly in SGIP1−/− and WT males, but fear extinction (FE) was more efficient in SGIP1−/− female mice compared to WT females. These results suggest that SGIP1 regulates anxiety levels in quite specific environments, possibly via modulation of CB1R signaling (Dvorakova et al., 2021).

We also examined acute responses and the development of tolerance to THC in the cannabinoid tetrad tests. After an initial treatment with THC, SGIP1−/− and WT mice exhibited similar levels of catalepsy, but tolerance to catalepsy developed significantly slower in SGIP1−/− mice. THC antinociception was also enhanced in the SGIP1−/− mice: latency was elevated in SGIP1−/− mice in the tail immersion test (TIT) *before* THC treatment, and SGIP1−/− mice showed greater analgesia to THC. Also, following 8 days of daily THC administration, tolerance to THC developed more slowly in SGIP1−/− males. Following chronic treatment with THC and development of THC dependence, rimonabant was applied to elicit a THC withdrawal syndrome. This resulted in abnormal jumping behaviors in SGIP1−/− mice, where jumping was more intense and persistent (Dvorakova et al., 2021). Interestingly, a similar jumping phenotype is prominent during morphine withdrawal (Francis and Schneider, 1971) and is decreased in CB1R−/− mice (Ledent et al., 1999).

CB1R activation decreases responses to painful stimuli (Hasanein et al., 2007; Mascarenhas et al., 2017; Woodhams et al., 2017). Compared to the WT animals, SGIP1−/− mice reaction latencies to acute nociception stimuli are increased, indicating that analgesia is increased at baseline. In the SGIP1−/− male cohort, deletion of SGIP1 synergistically enhanced THC and WIN antinociception in the TIT. One possible explanation for this result is crosstalk between the endocannabinoid system and other signaling pathways, specifically the opioid system (Canals and Milligan, 2008), also reviewed in (Robledo et al., 2008). There are synergistic-like effects between WIN and SGIP1 deletion. In the TIT, morphine produced different results in the SGIP1−/− mice compared with CB1R agonists. While SGIP1 deletion still enhanced morphine-induced antinociception, the interaction was weaker than for CB1R-mediated antinociception, consistent with an additive type of interaction between SGIP1 deletion and mu-opioid receptor activation (Dvorakova et al., 2021).

Insight into our study with SGIP1−/− mice can be gained by considering the results of behavioral tests from studies in which the ECS was manipulated chemically or genetically. Global deletion of CB1R causes an exploratory phenotype, hypoactivity, and anxiety-like behavior, particularly if CB1R−/− mice were examined under highly aversive conditions (Zimmer et al., 1999). The anxiolytic phenotype that was present in our studies of SGIP1−/− mice parallels the phenotype that is predicted following moderate upregulation of ECS tone.

Association of beta-arrestin2 with CB1R occurs following phosphorylation of serine and/or threonine residues in the C-terminus of the CB1R. Mice with a double mutation of two critical serine residues being exchanged to alanine residues CB1R (S426A, S430A) are more sensitive to THC (Morgan et al., 2014). Thus, the phenotype of CB1R (S426A, S430A) mice and SGIP1−/− mice overlap in their response to THC.

Manipulating endocannabinoid synthesis and degradation can profoundly influence behavior. Results from the behavioral testing of SGIP1−/− mice are reminiscent of mouse phenotypes observed after the manipulation of ECS signaling. Increasing anandamide levels via pharmacological inhibition of its degrading enzyme, fatty acid amide hydrolase (FAAH) (Kathuria et al., 2003), or genetic deletion of FAAH (Moreira et al., 2008) elicits phenotypes that are prominent for decreased anxiety-like behavior, as we observed in our evaluation of SGIP1−/− mice. On the other hand, global genetic deletion of diacylglycerol lipase alpha (DAGL alpha), the enzyme primarily responsible for neuronal 2-AG synthesis, increases levels of anxiety-like behavior (Shonesy et al., 2014; Jenniches et al., 2016). Another consideration is that altered CB1R signaling in the SGIP1−/− mice may affect related signaling cascades. Mice lacking beta-arrestin2 also have enhanced acute responses to THC, and tolerance is altered during chronic THC treatment (Breivogel et al., 2008; Nguyen et al., 2012). Genetic deletion of GASP1 reduces tolerance to cannabinoid-mediated antinociception in mice (Martini et al., 2010). Comparing results and synthesizing behavioral results from studies with mouse strains with manipulated levels of beta-arrestins or GASP1 have a high level of concordance with observations using SGIP1−/− mice.

Similarities between behaviors of genetically modified mouse strains in genes related to the ECS and SGIP1−/− mice, together with altered sensitivity of these mice to pharmacological manipulations, provide support for our hypothesis that SGIP1 regulates emotional behavior by “fine-tuning” CB1R signaling.

## Conclusion

The unique properties of CB1R in the neuronal axonal compartment have been described in several studies. Compared to the rates of internalization from the somatodendritic membranes, or from the surface of transfected heterologous cells, the enhanced stability of basal or activated CB1R on axonal membranes may contribute to its polar distribution in neurons. SGIP1 and CB1R interact with functional consequences *in vitro*. Hypothetically, SGIP1 association with CB1R might underlie the resistance of the receptor to CME in axons. The knowledge of each mechanism that is involved in CB1R signaling regulation is rapidly growing. Further studies will depict how these machineries influence each other, as the net result, the CB1R signaling modification, is likely dependent on their synchronization and/or coordination. Experiments *in vivo* further implicate that the SGIP1-CB1R relationship is physiologically relevant.

Much of the motivation to study the interactions between SGIP1 and CB1R was driven by a desire to increase the therapeutic usefulness of CB1R agonists by decreasing tolerance and/or increasing the non-intoxicating therapeutic window of THC or other CB1R agonists. A major conclusion from the studies discussed in this review is that SGIP1 deletion in mice enhances CB1R agonist responses and increases morphine analgesia, while slowing the onset of their tolerance to both classes of agonist.

The endocannabinoid signaling is deeply involved in regulating energy balance. Therefore, another therapeutic direction that may exploit the SGIP1-CB1R relationship is novel treatments for obesity. While the CB1R inverse agonist rimonabant proved to be an effective anti-obesity drug, undesired side effects, such as depression, resulted in its withdrawal from clinical use. Lack of physical activity combined with increased energy intake leads to obesity in humans. SGIP1 overproduction in the hypothalamus of the Israeli sand rat leads to obesity and metabolic syndrome, if the animals are kept in captivity with food *ad libitum*. Therefore, pharmacological attenuation of the SGIP1-CB1R association should be explored as a possible treatment for energy balance disorders.

These examples illustrate potential pharmacological approaches based on manipulating SGIP1-CB1R association to treat pain and obesity. Other pathological conditions, in which CB1R signaling modulation may be also beneficial include anxiety or other states involving the ECS.

## Author contributions

JB, KM: conceptualization. JB: writing—original draft preparation and funding acquisition. OD, KM, and JB: writing—review and editing. OD: visualization. All authors contributed to the article and approved the submitted version.

## Funding

This research was funded by Grant Agency of Czech Republic (19-24172S and 21-02371S).

## Conflict of interest

The authors declare that the research was conducted in the absence of any commercial or financial relationships that could be construed as a potential conflict of interest.

## Publisher’s note

All claims expressed in this article are solely those of the authors and do not necessarily represent those of their affiliated organizations, or those of the publisher, the editors and the reviewers. Any product that may be evaluated in this article, or claim that may be made by its manufacturer, is not guaranteed or endorsed by the publisher.
